# Evaluation of bone marrow-derived cell-based therapies in the hindlimb ischaemia model: a protocol for a systematic review and meta-analysis

**DOI:** 10.1136/bmjos-2021-100209

**Published:** 2021-12-16

**Authors:** Femke Christina Ching Chuan van Rhijn-Brouwer, Robin Wilhelmus Maria Vernooij, Kimberley Wever, Iris Schilt, Joos Ougust Fledderus, Maria Christina Verhaar, Hendrik Gremmels

**Affiliations:** 1 Department of Nephrology & Hypertension, Regenerative Medicine Center Utrecht, University Medical Center Utrecht, Utrecht, The Netherlands; 2 Department of Nephrology, University Medical Center Utrecht, Utrecht, The Netherlands; 3 Julius Center for Health Sciences and Primary Care, University Medical Center Utrecht, Utrecht, The Netherlands; 4 Radboud Institute for Health Sciences, Radboud University Medical Center, Nijmegen, The Netherlands

**Keywords:** cell therapy, hindlimb ischemia, angiogenesis, cardiovascular disease, peripheral artery disease

## Abstract

**Objective:**

Bone marrow(BM)-derived cell-based therapies for critical limb ischamia showed less clinical benefit than expected. While this might be due to patient-specific factors, it remains possible that important details were lost in the bench-to-clinic translation. The hindlimb ischaemia model is the golden standard to evaluate cell-based therapies aimed at promoting neovascularisation. To inform future trial design and identify potential knowledge gaps, we propose a systematic review and meta-analysis of preclinical evidence to assess the efficacy of BM-derived cell administration in restoring relative perfusion in the hind limb model and identify determinants of therapeutic efficacy.

**Search strategy:**

PubMed and EMBASE were searched for prospective studies in which the hindlimb ischaemia model was used to assess BM-derived therapies.

**Screening and annotation:**

Studies with an outcome measure related to relative perfusion of the hindlimb will be included. Study characteristics which include model-related factors as well as details on BM therapy will be extracted.

**Data management and reporting:**

For the primary analysis, a random effects model will be constructed using the mean difference calculated from the maximum relative perfusion for each study arm in each study. A separate model will be constructed using the relative perfusion at the latest time point in each study. We will also assess the risk of bias using the SYRCLE tool for internal validity. Subgroup analysis will be performed on animal characteristics, administration route, dose and cell characteristics such as the cell donor.

**PROSPERO registration number:**

This protocol has been registered at PROSPERO (CRD2021226592).

**Strengths and limitations of this study**

- No previous meta-analysis has been performed in this field. Comprehensive search strategy without limitations on language or publication date.
- Risk of bias analysis according to the most rigorous standards of the field.
- Predefined comprehensive subgroup analyses

## Introduction

In the last decades, bone marrow (BM)-derived cell-based therapies have been explored as a novel treatment option for patients with critical limb ischaemia (CLI). BM-derived therapies can improve vascularisation through paracrine secretion of pro-angiogenic growth factors, cytokines and extracellular vesicles.^1 2^ The Therapeutic Angiogenesis by Cell Transplantation (TACT) clinical trial in 2002 showed for the first time that autologous BM-derived cell therapy could be safe and effective for therapeutic neovascularisation in CLI^3^. Despite initial promising results, many of the larger randomised clinical studies using BM-derived cells to treat CLI did not show an advantage of cell therapy over placebo.^4 5^ The exact reason for this discrepancy is not clear, but possible explanations include differences in physiology of the trial participants, the cell types used or the route of administration.^6^ Human clinical trials on BM-derived cell therapies for CLI have been initiated very early in the process, based on only few preclinical studies—the TACT trial was published only 3 years after a rationale for cell therapy was proposed.^4 5^

Although preclinical research is an essential step in the development of clinically viable therapies, the clinical translation is often not without hurdles. In part, this may be due to the limitations of the (animal) models used, but also because the experimental design of preclinical research does not always support the design of clinical trials in humans.^7 8^ Presently, no systematic evaluation of the preclinical evidence preceding trials in patients has been performed. Meta-analysis of preclinical studies has been proposed as a method to inform clinical trial design and to elucidate why some very promising therapies fail clinical evaluation. While an individual study might not be sufficiently powered to favour one study design over another, for example, with regard to administration route, dose or cell type, meta-analysis of pooled data can reveal trends that otherwise would have gone unnoticed.^9^ We will, therefore, perform a systematic review and meta-analysis of the preclinical in vivo evidence base for the use of BM-derived cells in CLI. We will assess the available evidence and investigate how treatment-specific factors such as administration routes, doses and the timing affect therapeutic efficacy, which will inform future research in animals or humans.

The hindlimb ischaemia model, as first described by Couffinhal *et al*, is the primary model used for in vivo assessment of novel therapies aimed at vascular regeneration.^10^ In this model, the femoral artery of one hindlimb is ligated or banded. The consequent reduction in hindlimb blood flow can be assessed in various ways, most commonly laser Doppler perfusion imaging (LDPI). The potential effect of interventions can then be assessed by determining the change in perfusion.

Up until now, there has not been a comprehensive synthesis of evidence for BM cell-based therapies in the hind limb ischaemia model.

Here, we describe the protocol for the systematic review and meta-analysis. The main question of this review is: What is the efficacy of BM derived cell administration in restoring relative perfusion in the hind limb model? If an effect of BM derived stem cells is found, we will ask the following subquestion: What are the determinants of therapeutic efficacy? This question will be split in cell-based determinants such as cell type, administration route or cell dose, and donor/recipient determinants.

## Methods

### Protocol registration

This section is structured according to the Systematic Review Protocol for Animal Intervention Studies format^9^ (online supplemental file 1).

10.1136/bmjos-2021-100209.supp1 Supplementary data

We are aware that a protocol for a review with a similar research question was registered at PROSPERO on 8 May 2019 (CRD42019126308), which has not yet been published. We feel that our review complements and improves on the existing effort, because several key aspects of our review methodology differ: (1) we will perform an extensive comprehensive search, including the use of the SYstematic Review Center for Laboratory animal Experimentation (SYRCLE)’s search filters for animals studies, to maximise study retrieval, (2) we will not apply language restrictions to avoid reporting bias, (3) we focus on BM-derived cell types that have Good Manufacturing Practice (GMP)-approved production pathways, which will facilitate clinical application of our findings, (4), we will perform a rigorous assessment of study quality by using the SYRCLE risk of bias tool, which assesses the internal validity of studies, rather than reporting quality only and (5) we will use the GRADE framework for animal studies to assess the quality of evidence in this review.^11^

### Inclusion criteria

#### Study population

We will include in vivo studies in animals that underwent permanent femoral artery ligation or banding in one limb. Control animals are those who received no treatment at all, or were treated with placebo or vehicle (saline or cell culture medium). Control animals must be separate animals from the treatment group. As the outcome measure is relative perfusion, that is, perfusion of the ligated limb is expressed relative to that of the contralateral (non-ligated limb), contralateral limbs of treated cannot serve as treatment controls. There will be no restrictions on the species, age, biological sex or concomitant disease of the animals used in the study.

#### Intervention

The intervention is defined as administration of BM-derived mononuclear cells (BM MNCs) or BM-derived mesenchymal stromal cells (BM MSCs). We define BM MNCs as BM-derived cells that underwent no other manipulation than erythrocyte lysis or density gradient centrifugation. We define BM MSCs as tissue culture plastic-adherent cells, obtained from BM. While the International Society for Stem Cell Research maintains more detailed criteria for MSCs, including marker expression and trilineage differentiation,^12^ these do not fully translate across species^13^ and cells used in preclinical studies are often incompletely characterised. There will be no restrictions on administration route, dose, frequency or timing of administration post hind limb ischaemia induction.

#### Outcome

The primary outcome measure is relative perfusion as measured by LDPI, laser speckle contrast imaging or another technique that reports perfusion as fraction of perfusion in the ligated limb to that of the contralateral non-ligated limb.

#### Study designs

We will include prospective, controlled, intervention studies with separate treatment arms

### Exclusion criteria

1. Not a primary in vivo animal study.
2. No hind limb ischaemia model applied (eg, non-permanent methods of ligation).
3. No cellular product administered.
4. Cellular product other than unmodified BM MNC or BM MSC administered.
5. No relevant outcome measures reported.
6. Absence of an appropriate control.
7. All cohorts received cointerventions or comedications.
8. Full text not retrievable.

### Search strategy and definitions

PubMed and Embase will be searched using a comprehensive search strategy with the search components “limb ischemia”, “peripheral occlusive arterial disease”, “stem cells” and “animal” (see online supplemental figure 2 for the complete search string).

10.1136/bmjos-2021-100209.supp2 Supplementary data

### Study selection process

The search will be loaded into the CAMARADES/ NC3Rs Systematic Review Facility webtool. Studies will first be screened for eligibility on title/abstract by two independent researchers (FCCCvR-B and HG). In this first stage, all studies that include the hindlimb ischaemia model and a BM-derived cell-based treatment will be included. A third researcher (JOF) will adjudicate in case of discrepancies. In the second stage, final inclusion will be determined based on the full text.

There are no restrictions on language or publication date. Reference lists of included studies and relevant reviews will be screened for further eligible articles.

### Extraction of study characteristics and outcome data

Identifying information such as first author, year and journal will be extracted. The study-specific characteristics that will be extracted are listed in the appendix (items 31–35), and include characteristics related to the animal model used, the cellular therapy investigated and details on the outcome measures used. Important items in the context of the model include the animal species used and whether it was immunocompromised or not. Intervention characteristics include cell type used, cell dose, the number of administrations, the administration route and timing of administration.

Relative perfusion measured with LDPI or laser speckle contrast as ratio of ischaemic/non-ischaemic limb at all reported time points after ligation will be recorded. Means and accompanying SD or SE of the mean will be extracted, Data will be extracted from graphs when numerical data is not reported, using the freely available Graph Digitizer software.

When it is not reported whether error bars represent SD or SE, we will assume that it is the SE for a conservative estimate. If ranges are reported for group size, we will assume the lowest number.

Study characteristics and data will be extracted by one person. A random selection of 10% will be assessed by a second assessor to determine accuracy of the data extraction. In case of discrepancies which cannot be resolved by discussion between the two reviewers, a third assessor will mediate resolution.

### Risk of bias assessment

The risk of bias will be assessed by two independent reviewers using SYRCLE’s Risk of Bias Tool.^14^ In case of discrepancies which cannot be resolved by discussion between the two reviewers, a third assessor will mediate resolution.

### Data reporting and statistical analysis

All study data will be reported in a descriptive summary. A meta-analysis will be performed if we can include at least 10 individual studies in the overall analysis. The effect size will be reported as raw mean differences.

For the primary analysis, we will use a random effects model to pool the mean difference calculated from the time points with the maximum relative perfusion for each study arm in each study. As a sensitivity analysis, we will rerun the analysis using the latest time point in each study. Heterogeneity will be assessed using the I^2^. Subgroup analyses will be performed if there are at least five studies per stratum of a given variable. Planned meta-regression subgroup analyses are listed in the appendix (item 47). They include animal-specific characteristics, such as species/background, immunocompetency, additional cardiovascular disease, as well as therapy characteristics, such as the origin of the cells, the administration route and the dose. Correction for multiple testing will be applied using the Bonferroni-Holmes method.

### Publication bias

Overall publication bias will be assessed by visual inspection of the funnel plot, as well as trim-and-fill analysis to assess funnel plot asymmetry. A minimum of 10 studies is needed (see above).

### Sensitivity analyses

As mentioned above, we will conduct a sensitivity analysis regarding the chosen time points (maximum effect vs latest time point). Additional sensitivity analyses are not yet planned.

### GRADE assessment

We will use the draft GRADE4animals framework^11^ to assess and rate the quality of the evidence per outcome in terms of risk of bias, indirectness (two levels), imprecision, inconsistency and publication bias.

## Discussion

Regenerative strategies have a high potential as prospective therapy in vascular disease, but recent negative trial results have increased the demand for solid preclinical evidence.^15^ Currently, the HLI model is the golden standard for assessing therapies aimed at promoting neovascularisation, even though it is not known whether the degree of restoration of perfusion in the HLI model directly correlates with the degree of clinical success.

With this synthesis of evidence, we will gain more insight in the effect sizes to be expected and the reliability and validity of the studies conducted. Additionally, our subgroup analyses will elucidate the various determinants of therapeutic efficacy. By carefully and thoroughly assessing the current body of evidence we expect to inform future trial design and bring evidence-based regenerative therapies closer to the clinic.

## References

[1] Watt SM, Gullo F, van der Garde M, et al. The angiogenic properties of mesenchymal stem/stromal cells and their therapeutic potential. Br Med Bull 2013;108:25–53. 10.1093/bmb/ldt031 24152971 PMC3842875

[2] Lai RC, Arslan F, Lee MM, et al. Exosome secreted by MSC reduces myocardial ischemia/reperfusion injury. Stem Cell Res 2010;4:214–22. 10.1016/j.scr.2009.12.003 20138817

[3] Tateishi-Yuyama E, Matsubara H, Murohara T, et al. Therapeutic angiogenesis for patients with limb ischaemia by autologous transplantation of bone-marrow cells: a pilot study and a randomised controlled trial. Lancet 2002;360:427–35. 10.1016/S0140-6736(02)09670-8 12241713

[4] Peeters Weem SMO, Teraa M, de Borst GJ, et al. Bone marrow derived cell therapy in critical limb ischemia: a meta-analysis of randomized placebo controlled trials. Eur J Vasc Endovasc Surg 2015;50:775–83. 10.1016/j.ejvs.2015.08.018 26460286

[5] Rigato M, Monami M, Fadini GP. Autologous cell therapy for peripheral arterial disease: systematic review and meta-analysis of randomized, nonrandomized, and Noncontrolled studies. Circ Res 2017;120:1326–40. 10.1161/CIRCRESAHA.116.309045 28096194

[6] Teraa M, Gremmels H, Wijnand JGJ, et al. Cell therapy for chronic limb-threatening ischemia: current evidence and future directions. Stem Cells Transl Med 2018;7:842–6. 10.1002/sctm.18-0025 30070050 PMC6265636

[7] Hooijmans CR, de Vries RBM, Rovers MM, et al. The effects of probiotic supplementation on experimental acute pancreatitis: a systematic review and meta-analysis. PLoS One 2012;7:e48811. 10.1371/journal.pone.0048811 23152810 PMC3496732

[8] Paauw ND, Terstappen F, Ganzevoort W, et al. Sildenafil during pregnancy: a preclinical meta-analysis on fetal growth and maternal blood pressure. Hypertension 2017;70:998–1006. 10.1161/HYPERTENSIONAHA.117.09690 28893896

[9] de Vries RBM, Wever KE, Avey MT, et al. The usefulness of systematic reviews of animal experiments for the design of preclinical and clinical studies. Ilar J 2014;55:427–37. 10.1093/ilar/ilu043 25541545 PMC4276599

[10] Couffinhal T, Silver M, Zheng LP, et al. Mouse model of angiogenesis. Am J Pathol 1998;152:1667–79. 9626071 PMC1858441

[11] Hooijmans CR, de Vries RBM, Ritskes-Hoitinga M, et al. Facilitating healthcare decisions by assessing the certainty in the evidence from preclinical animal studies. PLoS One 2018;13:e0187271. 10.1371/journal.pone.0187271 29324741 PMC5764235

[12] Dominici M, Le Blanc K, Mueller I, et al. Minimal criteria for defining multipotent mesenchymal stromal cells. The International Society for cellular therapy position statement. Cytotherapy 2006;8:315–7. 10.1080/14653240600855905 16923606

[13] Uder C, Brückner S, Winkler S, et al. Mammalian MSC from selected species: features and applications. Cytometry A 2018;93:32–49. 10.1002/cyto.a.23239 28906582

[14] Hooijmans CR, Rovers MM, de Vries RBM, et al. SYRCLE's risk of bias tool for animal studies. BMC Med Res Methodol 2014;14:43. 10.1186/1471-2288-14-43 24667063 PMC4230647

[15] Taylor-Weiner H, Graff Zivin J. Medicine's Wild West--Unlicensed Stem-Cell Clinics in the United States. N Engl J Med 2015;373:985–7. 10.1056/NEJMp1504560 26352811

