# Supplementary material for: Evaluation of bone marrow-derived cell-based therapies in the hindlimb ischaemia model: a protocol for a systematic review and meta-analysis
Source: BMJ Open Sci. 2021 Dec 16;5(1):e100209. doi: 10.1136/bmjos-2021-100209 (PMC8749269; doi:10.1136/bmjos-2021-100209)
Supplement: Supplementary data [file bmjos-2021-100209supp001.pdf]

SYSTEMATIC REVIEW PROTOCOL FOR ANIMAL INTERVENTION STUDIES

FORMAT BY SYRCLE (WWW.SYRCLE.NL)
VERSION 2.0 (DECEMBER 2014)

Item #	Section/Subsection/Item	Description	Check for approval
	A. General		
1.	Title of the review	The hindlimb ischemia model to assess the efficacy of cellular therapy for ischemic vascular disease	
2.	Authors (names, affiliations, contributions)	Femke van Rhijn. University Medical Center Utrecht<br>Dr Robin Vernooij. University Medical Center Utrecht<br>Dr Joost Fledderus. University Medical Center Utrecht<br>Dr Hendrik Gremmels. University Medical Center Utrecht<br>Dr Marianne Verhaar. University Medical Center Utrecht<br>Dr Kim Wever. SYRCLE, Radboudumc	
3.	Other contributors (names, affiliations, contributions)	Iris Schilt – master student<br>Dr Kim Wever. SYRCLE, Radboudumc	
4.	Contact person + e-mail address	m.c.verhaar@umcutrecht.nl	
5.	Funding sources/sponsors	The Netherlands Organization for Health Research and Development, Meer Kennis met Minder Dieren programma (More Knowledge using Fewer Animals programme)	
6.	Conflicts of interest	None	
7.	Date and location of protocol registration	PROSPERO	
8.	Registration number (if applicable)	PROSPERO 2021 CRD42021226592	
9.	Stage of review at time of registration		
	B. Objectives		
	Background		
10.	What is already known about this disease/model/intervention? Why is it important to do this review?	Despite initial promising results, many of the larger randomised clinical studies utilizing bone marrow (BM) derived cells to treat critical limb ischemia did not show an advantage of cell therapy over placebo. The exact reason for this discrepancy is not clear.<br><br>The hindlimb ischemia model is commonly used to assess regenerative treatments for peripheral artery disease. We therefore aim to conduct this review to collate all the preclinical evidence for bone marrow-derived cell-based interventions in peripheral artery disease.	
	Research question		
11.	Specify the disease/health problem of interest	Peripheral artery disease	
12.	Specify the population/species studied	Any animal model of hindlimb ischemia by ligation or other permanent form of disruption of the continuity of the blood supply to the lower limb.	
13.	Specify the intervention/exposure	Administration of bone marrow derived mononuclear cells or bone marrow derived mesenchymal stromal cells	
14.	Specify the control population	Vehicle injection or no treatment	

15.	Specify the outcome measures	Relative perfusion of the limb (after intervention)	
16.	State your research question (based on items 11-15)	What is the effect of bone marrow derived cell products on limb perfusion in animal models of hindlimb ischemia?<br><br>Sub-questions: what is the most effective cell type, cell dose and administration route for regenerative treatment in limb ischemia?	
	C. Methods		
	Search and study identification		
17.	Identify literature databases to search (*e.g.* Pubmed, Embase, Web of science)	X MEDLINE via PubMed ☐Web of Science<br>☐SCOPUS XEMBASE<br>☐Other, namely:<br>☐Specific journal(s), namely:	
18.	Define electronic search strategies (*e.g.* use the step by step search guide[15] and animal search filters[20, 21])	See supplementary file 2 for the search strategy	
19.	Identify other sources for study identification	XReference lists of included studies ☐Books<br>XReference lists of relevant reviews<br>☐Conference proceedings, namely:<br>☐Contacting authors/ organisations, namely:<br>☐Other, namely:	
20.	Define search strategy for these other sources	We will screen the reference lists of both relevant reviews (identified through our search strategy) and included studies, for additional references to possibly relevant studies. These references will selected on title and are then added to the screening process, and screened as usual (described under 21-21)	
	Study selection		
21.	Define screening phases (*e.g.* pre-screening based on title/abstract, full text screening, both)	1). Screening on eligibility on Title/Abstract<br>2). Screening on final inclusion on full text	
22.	Specify (a) the number of reviewers per screening phase and (b) how discrepancies will be resolved	Title/abstract: 2 reviewers.<br>Full text screening: 1 reviewer.<br>Discrepancies will be resolved through discussion, a third reviewer will act as tie-breaker.	
	Define all inclusion and exclusion criteria based on:		
23.	Type of study (design)	Inclusion criteria: Prospective, controlled, intervention study with separate treatment arms<br>Exclusion criteria: Case reports, Observational studies, Cross-over studies, non primary studies such as reviews	
24.	Type of animals/population (*e.g.* age, gender, disease model)	Inclusion criteria: Animals undergoing femoral artery ligation or banding in one limb (co-morbidities and knock-outs incl. immunosuppressed animals will annotated as covariable) .<br>Exclusion criteria: Studies in humans, in vitro or in silico studies. Non-permanent disruption of blood flow (such as ischemia-reperfusion models that feature a temporary	

		ligation)	
25.	Type of intervention (*e.g.* dosage, timing, frequency)	Inclusion criteria: Administration of bone marrow derived mononuclear cells or bone marrow derived mesenchymal stromal cells (any route, dose or timing) or vehicle control / no treatment<br>Exclusion criteria: No administration of a cellular product, (such as conditioned medium) additional manipulations such as sorting or bead-selection of the cells	
26.	Outcome measures	Inclusion criteria: relative perfusion as measured with laser doppler imaging or laser speckle imaging<br>Exclusion criteria: No relevant outcome measures reported	
27.	Language restrictions	Inclusion criteria: All<br>Exclusion criteria: None	
28.	Publication date restrictions	Inclusion criteria: all dates<br>Exclusion criteria: None	
29.	Other		
30.	Sort and prioritize your exclusion criteria per selection phase	Title and abstract:<br>1) Not a primary in vivo animal study<br>2) No hind limb ischemia model applied<br>3) No cellular product administered<br><br>Full-text screening:<br>1) Not a primary *in vivo* animal study<br>2) No hind limb ischemia model applied (e.g. non-permanent methods of ligation)<br>3) No cellular product administered<br>4) Cellular product other than unmodified BM MNC or BM MSC administered<br>5) No relevant outcome measures reported<br>6) Absence of an appropriate control<br>7) All cohorts received co-interventions or co-medications<br>8) Full-text not retrievable	
	Study characteristics to be extracted (for assessment of external validity, reporting quality)		
31.	Study ID (*e.g.* authors, year)	First author<br>Year<br>Journal<br>PMID	
32.	Study design characteristics (*e.g.* experimental groups, number of animals)	Experimental groups<br>Number of animals per experimental group	
33.	Animal model characteristics (*e.g.* species, gender, disease induction)	• Species<br>• Sex<br>• Age at study start<br>• Additional disease (e.g. DM)<br>• Immunocompromised yes/no	

34.	Intervention characteristics (*e.g.* intervention, timing, duration)	• Treatment<br>• Control type<br>• Recorded Timepoints<br>• Duration of study (post HLI induction)<br>• Timing of intervention post hindlimb ischemia induction<br>• Number of administrations (+ timing of second/third etc administration)<br>• Dose<br>• Administration route<br>• Administration location if available<br><br>Details on cells used:<br>• Cell type<br>• Donor Species<br>• Allogenic yes/no<br>• Sex of donor m/f<br>• Age of donor<br>• Cyropreservation yes/no	
35.	Outcome measures	Timing of the outcome measurement in hours post induction of hindlimb ischemia<br><br>Relative perfusion as measured by laser Doppler perfusion imaging or laser speckle contrast analysis (this is reported as a ratio of the ischemic:non ischemic leg).	
36.	Other (*e.g.* drop-outs)		
	Assessment risk of bias (internal validity) or study quality		
37.	Specify (a) the number of reviewers assessing the risk of bias/study quality in each study and (b) how discrepancies will be resolved	2 reviewers. Discrepancies will be resolved through discussion.	
38.	Define criteria to assess (a) the internal validity of included studies (*e.g.* selection, performance, detection and attrition bias) and/or (b) other study quality measures (*e.g.* reporting quality, power)	X By use of SYRCLE's Risk of Bias tool[4]<br>☐ By use of SYRCLE's Risk of Bias tool, adapted as follows:<br>☐ By use of CAMARADES' study quality checklist, e.g [22]<br>☐ By use of CAMARADES' study quality checklist, adapted as follows:	
	Collection of outcome data		
39.	For each outcome measure, define	Primary outcome	

	the type of data to be extracted (*e.g.* continuous/dichotomous, unit of measurement)	Relative perfusion<br>[continuous]: reported as fraction	
40.	Methods for data extraction/retrieval (*e.g.* first extraction from graphs using a digital screen ruler, then contacting authors)	1. Copying reported values if mentioned in paper (visual check whether it is similar to graphs)<br>2. Extraction from graphs using Plot digitizer	
41.	Specify (a) the number of reviewers extracting data and (b) how discrepancies will be resolved	1 reviewer will extract the data. A random selection of 10% will be assessed by a second assessor to determine accuracy of the data extraction. A third assessor will mediate resolution.	
	Data analysis/synthesis		
42.	Specify (per outcome measure) how you are planning to combine/compare the data (*e.g.* descriptive summary, meta-analysis)	All outcome data will be reported in a descriptive summary which will include animal characteristics, treatment characteristics, and details on the cells used. A meta analysis will be conducted if at least 10 studies can be included in the analysis. Subgroup analyses will be performed if at least 5 studies can be included in each stratum. Correction for multiple testing will be applied using the Bonferroni-Holmes method.	
43.	Specify (per outcome measure) how it will be decided whether a meta-analysis will be performed	relative perfusion: We require at least 10 individual studies in the overall analysis. For sub-group analyses, at least 5 studies per stratum.	
	If a meta-analysis seems feasible/sensible, specify (for each outcome measure):		
44.	The effect measure to be used (*e.g.* mean difference, standardized mean difference, risk ratio, odds ratio)	Relative perfusion: Mean difference	
45.	The statistical model of analysis (*e.g.* random or fixed effects model)	Random effects model	
46.	The statistical methods to assess heterogeneity (*e.g.* I^2, Q)	I^2	
47.	Which study characteristics will be examined as potential source of heterogeneity (subgroup analysis)	Animal characteristics:<br>• Species<br>• Additional cardiovascular disease<br>• Immunocompromised animals<br><br>Intervention characteristics:<br>• Administration route<br>• Dose<br><br>Details on the cells used:<br>• Cell type<br>• Donor Species<br>• Allogenic/Xenogeneic<br>• Diseased Donor<br>• Cryopreservation	C

48.	Any sensitivity analyses you propose to perform	As a sensitivity analysis, we will re-run the analysis using the latest time point in each study (as opposed to the maximum perfusion)	
49.	Other details meta-analysis (*e.g.* correction for multiple testing, correction for multiple use of control group)	The Bonferroni-Holmes correction will be applied to correct for multiple testing.	
50.	The method for assessment of publication bias	Publication bias will be assessed over the overall effect by funnel plot. A minimum of 10 studies is needed (see above). The trim-and-fill test will be used to assess asymmetry.	
Final approval by (names, affiliations):		Date:	
