# Supplementary material for: Evaluation of bone marrow-derived cell-based therapies in the hindlimb ischaemia model: a protocol for a systematic review and meta-analysis
Source: BMJ Open Sci. 2021 Dec 16;5(1):e100209. doi: 10.1136/bmjos-2021-100209 (PMC8749269; doi:10.1136/bmjos-2021-100209)
Supplement: Supplementary data [file bmjos-2021-100209supp002.pdf]

Supplementary file 2: search strategy

PubMed		
#1	Ischemia	ischemia[Mesh] OR "warm ischemia"[Mesh] OR “cold ischemia”[Mesh] OR ischemia[tiab] OR ischaemia[tiab] OR ischemic[tiab] OR ischaemic[tiab] OR ischemically[tiab] OR ischaemically[tiab] OR postischemic[tiab] OR postischaemic[tiab] OR “post-ischemic”[tiab] OR “post-ischaemic”[tiab]
#2	Limb	extremities[Mesh] OR hindlimb[Mesh] OR extremity[tiab] OR extremities[tiab] OR limb[tiab] OR limbs[tiab] OR hindlimb[tiab] OR hindlimbs[tiab] OR forelimb[tiab] OR forelimbs[tiab] OR leg[tiab] OR legs[tiab] OR foot[tiab] OR feet[tiab] OR hindleg[tiab] OR hindlegs[tiab] OR foreleg[tiab] OR forelegs[tiab] OR arm[tiab] OR arms[tiab] OR paw[tiab] OR paws[tiab] OR hindpaw[tiab] OR hindpaws[tiab] OR forepaw[tiab] OR forepaws[tiab]
#3	Limb AND ischemia	(#1 AND #2)
#4	PAOD	"Peripheral Arterial Occlusive Disease 1" [Supplementary Concept] OR "Thromboangiitis Obliterans"[Mesh] OR “femoral artery ligation”[tiab] OR “femoral artery primary ligation”[tiab] OR “femoral arterial ligation”[tiab] OR “iliac artery ligation”[tiab] OR “iliac arterial ligation”[tiab] OR “peripheral arterial disease”[tiab] OR “peripheral arterial diseases”[tiab] OR “peripheral artery disease”[tiab] OR “peripheral artery diseases”[tiab] OR “peripheral arterial occlusi*”[tiab] OR “peripheral artery occlusi*”[tiab] OR “peripheral arterial obliterati*”[tiab] OR “peripheral artery obliterati*”[tiab] OR “peripheral arterial obstructi*”[tiab] OR “peripheral artery obstructi*”[tiab] OR PAOD[tiab] OR “peripheral occlusive arterial disease”[tiab] OR “peripheral occlusive arterial diseases”[tiab] OR “peripheral obstructive arterial disease”[tiab] OR “peripheral obstructive arterial diseases”[tiab] OR “peripheral obliterative arterial disease”[tiab] OR “peripheral obliterative arterial diseases”[tiab] OR “peripheral occlusive artery disease”[tiab] OR “peripheral occlusive artery diseases”[tiab] OR “peripheral obstructive artery disease”[tiab] OR “peripheral obstructive artery diseases”[tiab] OR “peripheral obliterative artery disease”[tiab] OR “peripheral obliterative artery diseases”[tiab] OR POAD[tiab] OR “arteriosclerosis obliterans”[tiab] OR “atherosclerosis obliterans”[tiab] OR “peripheral arteriosclerotic occlusion”[tiab] OR “peripheral atherosclerotic occlusion”[tiab] OR “peripheral arteriolerotic occlusions”[tiab] OR “peripheral atherosclerotic occlusions”[tiab] OR “peripheral arterial insufficiency”[tiab] OR “peripheral arterial insufficiencies”[tiab] OR “peripheral artery insufficiency”[tiab] OR “peripheral artery insufficiencies”[tiab] OR “thromboangitis obliterans”[tiab] OR Buerger*[tiab] OR “ischemic ulcer”[tiab] OR “ischaemic ulcer”[tiab] OR “ischemic ulcers”[tiab] OR “ischaemic ulcers”[tiab] OR gangrene[tiab]
#5	(limb AND ischemia) OR PAOD	#3 OR #4
#6	stem cells	("stem cells"[MeSH] OR "Stem Cell Research"[Mesh] OR "Stem Cell Transplantation"[Mesh] OR "Bone Marrow Cells"[Mesh] OR "stem cell"[tiab] OR "stem cells"[tiab] OR "stromal cell"[tiab] OR "stromal cells"[tiab] OR "progenitor cell"[tiab] OR "progenitor cells"[tiab] OR “precursor cell”[tiab] OR “precursor cells”[tiab] OR "mother cell"[tiab] OR "mother cells"[tiab] OR "cell therapy"[tiab] OR "cell therapies"[tiab] OR

		"cell-based therapy"[tiab] OR "cellbased therapy"[tiab] OR "cell-based therapies"[tiab] OR "cellbased therapies"[tiab] OR "cell-based treatment"[tiab] OR "cellbased treatment"[tiab] OR "cell-based treatments"[tiab] OR "cellbased treatments"[tiab] OR MSC[tiab]) OR (("bone marrow"[tiab] OR bonemarrow[tiab] OR omnipotent[tiab] OR pluripotent[tiab]) AND (cell[tiab] OR cells[tiab]))	
#7	animals	Laboratory animal search filter [1]	
#8	limb ischemia OR PAOD AND stem cells AND animals		#5 AND #6 AND #7 NOT “review”[ptyp]

EMBASE		
#1	Ischemia	ischemia/ or experimental ischemia/ or exp peripheral ischemia/ or exp cold ischemia/ or exp muscle ischemia/ OR (ischemia OR ischaemia OR ischemic OR ischaemic OR ischemically OR ischaemically OR postischemic OR postischaemic OR post-ischemic OR post-ischaemic).ti,ab.
#2	Limb	exp limb/ OR (extremity OR extremities OR limb OR limbs OR hindlimb OR hindlimbs OR forelimb OR forelimbs OR leg OR legs OR foot OR feet OR hindleg OR hindlegs OR foreleg OR forelegs OR arm OR arms OR paw OR paws OR hindpaw OR hindpaws OR forepaw OR forepaws).ti,ab.
#3	Limb AND ischemia	(#1 AND #2)
#4	PAOD	exp limb ischemia/ OR exp critical limb ischemia/ OR exp leg ischemia/ OR peripheral occlusive artery disease/ OR blood vessel occlusion/ OR exp Buerger disease/ OR limb blood flow/ OR (femoral artery ligation OR femoral artery primary ligation OR femoral arterial ligation OR iliac artery ligation OR iliac arterial ligation OR peripheral arterial disease OR peripheral arterial diseases OR peripheral artery disease OR peripheral artery diseases OR peripheral arterial occlusi* OR peripheral artery occlusi* OR peripheral arterial obliterati* OR peripheral artery obliterati* OR peripheral arterial obstructi* OR peripheral artery obstructi* OR PAOD OR peripheral occlusive arterial disease OR peripheral occlusive arterial diseases OR peripheral obstructive arterial disease OR peripheral obstructive arterial diseases OR peripheral obliterative arterial disease OR peripheral obliterative arterial diseases OR peripheral occlusive artery disease OR peripheral occlusive artery diseases OR peripheral obstructive artery disease OR peripheral obstructive artery diseases OR peripheral obliterative artery disease OR peripheral obliterative artery diseases OR POAD OR arteriosclerosis obliterans OR atherosclerosis obliterans OR peripheral arteriosclerotic occlusion OR peripheral atherosclerotic occlusion OR peripheral arteriolerotic occlusions OR peripheral atherosclerotic occlusions OR peripheral arterial insufficiency OR peripheral arterial insufficiencies OR peripheral artery insufficiency OR peripheral artery insufficiencies OR thromboangitis obliterans OR Buerger* OR ischemic ulcer OR ischaemic ulcer OR ischemic ulcers OR ischaemic ulcers OR gangrene).ti,ab.
#5	(limb AND ischemia) OR PAOD	#3 OR #4

#6	stem cells	exp stem cell/ OR exp stem cell research/ OR exp stem cell transplantation/ OR exp bone marrow cell/ OR (stem cell OR stem cells OR stromal cell OR stromal cells OR progenitor cell OR progenitor cells OR precursor cell OR precursor cells OR mother cell OR mother cells OR cell therapy OR cell therapies OR cell-based therapy OR cellbased therapy OR cell-based therapies OR cellbased therapies OR cell-based treatment OR cellbased treatment OR cell-based treatments OR cellbased treatments OR MSC).ti,ab. OR ((bone marrow OR bonemarrow OR omnipotent OR pluripotent).ti,ab. AND (cell OR cells).ti,ab.)	
#7	animals	Laboratory animal search filter [2]	
#8	limb ischemia OR PAOD AND stem cells AND animals		#5 AND #6 AND #7
	limit 8 to (article or article in press or conference paper)		
